# Network-based transcranial direct current stimulation enhances attention function in healthy young adults: a preliminary study

**DOI:** 10.3389/fnhum.2024.1421230

**Published:** 2024-08-08

**Authors:** Xiaoyu Wei, Rong Zhou, Suwang Zheng, Yufeng Zhang, Xiaofan Feng, Jiaojiao Lü

**Affiliations:** ^1^Key Laboratory of Exercise and Health Sciences of Ministry of Education, Shanghai University of Sport, Shanghai, China; ^2^School of Exercise and Health, Shanghai University of Sport, Shanghai, China

**Keywords:** transcranial direct current stimulation, brain network modulation, attention, dorsal attention network, default network

## Abstract

**Purpose:**

Attention, a complex cognitive process, is linked to the functional activities of the brain’s dorsal attention network (DAN) and default network (DN). This study aimed to investigate the feasibility, safety, and blinding efficacy of a transcranial direct current stimulation (tDCS) paradigm designed to increase the excitability of the DAN while inhibiting the DN (DAN+/DN-tDCS) on attention function in healthy young adults.

**Methods:**

In this randomized controlled experiment, participants were assigned to either the DAN+/DN-tDCS group or the sham group. A single intervention session was conducted at a total intensity of 4 mA for 20 min. Participants completed the Attention Network Test (ANT) immediately before and after stimulation. Blinding efficacy and adverse effects were assessed post-stimulation.

**Results:**

Forty participants completed the study, with 20 in each group. Paired-sample t-test showed a significant post-stimulation improvement in executive effect performance (*t* = 2.245; *p* = 0.037) in the DAN+/DN-tDCS group. The sham group did not exhibit any significant differences in ANT performance. Participants identified the stimulation type with 52.50% accuracy, indicating no difference in blinding efficacy between groups (*p* = 0.241). Mild-to-moderate adverse effects, such as stinging, itching, and skin reddening, were reported in the DAN+/DN-tDCS group (*p* < 0.05).

**Conclusion:**

DAN+/DN-tDCS enhanced attention function in healthy young individuals, particularly in improving executive effect performance. This study presents novel strategies for enhancing attentional performance and encourages further investigation into the mechanisms and outcomes of these interventions across diverse populations.

## 1 Introduction

Attention is a critical component of human cognitive functions, involving the collection filtering, selecting, and processing of information from the external world, allowing us to focus on the most relevant and useful details (Buschman and Miller, 2007). Attention processes are supported by complex neural networks that interconnect and synchronize the activity of multiple brain regions, including the dorsal attention network (DAN) and the default network (DN) (Xuan et al., 2016; Zhao et al., 2021). The DAN exhibits heightened activity during tasks requiring external attention, facilitating the optimal allocation of cognitive resources and the prolonged maintenance of attention (Esterman et al., 2014; Dixon et al., 2017). Conversely, the DN, known as a task-negative correlation network, is typically suppressed during attention-demanding tasks but becomes more active during internal orientation and self-referential processes (Boord et al., 2017). The negatively correlated connectivity between the DAN and DN reflects the brain’s ability to switch between external attention and internally focused thought processes (Lo et al., 2021). This dynamic interconnectivity is essential for effectively allocating and maintaining attention (Esterman et al., 2014). Research has demonstrated that the stronger negative correlation between DAN and DN is related to highly productive cognitions (Kelly et al., 2008; Xu et al., 2015; Parkinson et al., 2019). Therefore, we hypothesized that enhancing the excitability of the DAN while concurrently suppressing the DN to coordinate the brain functional activities may effectively modulate individual attention function.

Transcranial direct current stimulation (tDCS) is a non-invasive and well-tolerated brain stimulation technique that induces significant changes in neuronal excitability by applying low-intensity (0.1 to 2.0 mA) anodal or cathodal stimulation to a target area (Paulus, 2003; Bikson et al., 2016). tDCS can modulate the excitability of the cerebral cortex, thereby enhancing the corresponding cortical motor or cognitive functions (Nitsche and Paulus, 2000; Trudgen et al., 2019). However, meta-analyses have concluded that there is insufficient evidence to assert that traditional tDCS significantly enhances attention (Majdi et al., 2022; Saleh et al., 2023). Previous research has applied traditional tDCS to target single brain regions, including the left dorsolateral prefrontal cortex (DLPFC) (Miler et al., 2018), the right posterior parietal cortex (PPC) (Lo et al., 2019) and the right prefrontal cortex (Coffman et al., 2014). These studies suggest that while traditional tDCS may improve attention to some extent, its effects on different aspects of attention are inconsistent. One explanation is that traditional tDCS, which focuses on a single target region, may not sufficiently enhance brain functional activities (Roy et al., 2015; Miler et al., 2018; Lu et al., 2020; Nawani et al., 2023). During attention processes, brain regions do not operate in isolation; rather, attention relies on the integration and collaboration of large-scale brain networks (Hutchison et al., 2013; Fiebelkorn and Kastner, 2020).

To address the need for brain network modulation, researchers have proposed a network-based tDCS model that combines specific algorithms to determine the optimal cortical electric fields and a set of optimized stimulation parameters. This technique utilizes simultaneous or coordinated stimulation of multiple task-related brain regions to alter excitability, enhancing connectivity in key cortical regions and amplifying the stimulation effect (Ruffini et al., 2014; Fischer et al., 2017; Mencarelli et al., 2020). Compared to traditional tDCS, network-based tDCS has been shown to have superior excitatory and inhibitory effects on brain networks (Mencarelli et al., 2020; Gregoret et al., 2023). For example, Zhou et al. (2022) demonstrated that network-based tDCS significantly reduced gait variability in healthy adults using a protocol known as DAN+/DN-tDCS, which is designed to simultaneously enhance DAN excitability while inhibiting DN excitability. While this approach theoretically offers potential benefits, its effects on attention function have not yet been clearly established.

Therefore, this randomized controlled trial aimed to investigate the feasibility and safety of the DAN+/DN-tDCS on attention function in healthy young adults. We hypothesized that this novel tDCS technique, designed to enhance coordination between the DAN and DN brain networks, would improve attention function. Additionally, we anticipated that this stimulation protocol would be safe without severe adverse effects.

## 2 Methods

### 2.1 Participants

This study recruited healthy young adults to assess the comfort and adverse effects associated with the stimulation protocols, which can offer better application to clinical populations. Participants had to be right-handed, as determined by the Edinburgh Handedness Inventory (Oldfield, 1971), possess normal or corrected vision, and have no history of neurological or psychological problems.

Exclusion criteria included: 1) hospitalization in the previous 6 months, 2) use of medications affecting brain states through central nervous system activity and neural activation, 3) severe neurological disorders (such as Parkinson’s disease and stroke), musculoskeletal conditions leading to gait impairment, or cerebrovascular and cardiovascular illnesses, 4) cognitive impairment (Mini-Mental Status Exam ≤24) (Arevalo-Rodriguez et al., 2021), and 5) any contraindication to tDCS stimulation (intracerebral metal implants) (Zhou et al., 2014).

All participants were required to have a basic understanding of network-based tDCS, comprehend the study objectives and procedures, and signed a written informed consent before testing. The study received ethical approval from the Ethics Committee of Shanghai University of Sports (102772020RT109).

### 2.2 Experimental protocol

**Figure 1:**
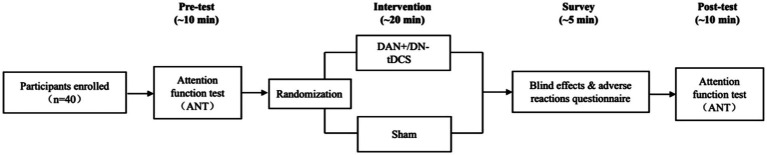
Experimental protocol. Participants performed the Attention Network Test (ANT) before and after DAN+/DN-tDCS or sham stimulation. DAN, dorsal attention network; DN, default network; tDCS, transcranial direct current stimulation.

Each enrolled participant attended the laboratory once for the experiment. Prior to the intervention, they underwent an Attention Network Test (ANT). Participants were randomly assigned to either the DAN+/DN-tDCS group or the sham group. Each group received a single 20-min intervention. Immediately after the intervention, participants completed another ANT and were provided a blinding efficacy and adverse event questionnaire (Figure 1). To minimize potential influences on attention function, participants refrained from intense physical activity for 24 h and from consuming caffeine-containing beverages for 4 h before the test.

### 2.3 Network-based tDCS

**Figure 2:**
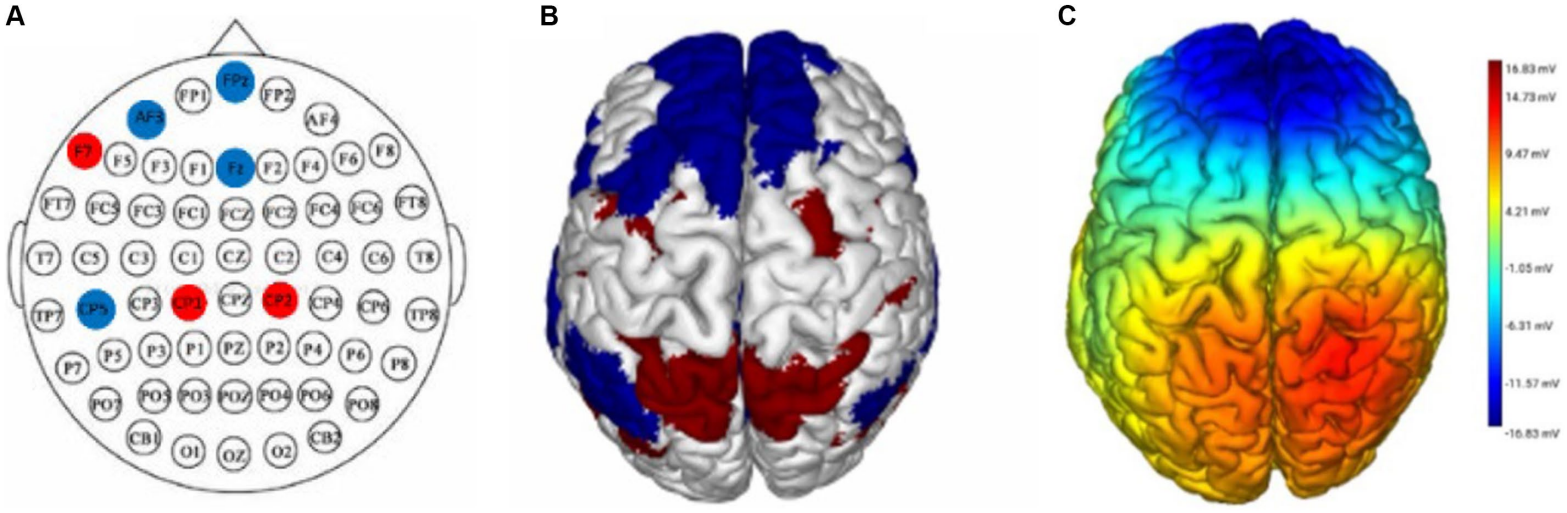
DAN+/DN-tDCS intervention, stimulation targets, and electric field modeling. **(A)** the electrode placement for the DAN+/DN-tDCS protocol, with red circles indicating anodes and blue circles indicating cathodes; **(B)** the red and blue parts represent the areas where the DAN and DN are located, respectively; **(C)** simulation of the electric field for the DAN+/DN-tDCS protocol and the color represents the current intensity; darker color indicates a greater current. DAN, dorsal attention network; DN, default network; tDCS, transcranial direct current stimulation.

This experiment employed the Neuroelectrics Stimweaver^®^ platform to implement the DAN+/DN-tDCS and sham stimulation protocols. For this purpose, a 3.14 cm^2^ Ag/AgCl gel electrode was positioned within a neoprene cap at a location specified by the International 10–20 electroencephalography system (Ulate-Campos and Loddenkemper, 2024). The DAN+/DN-tDCS protocol involved placing seven electrodes on key regions of the DAN and DN based on the international 10–20 EEG system. Anode electrodes were situated at F7, CP1, and CP2, while cathode electrodes were placed at FP_Z_, F_Z_, CP5, and AF3. This arrangement aimed to excite the DAN and inhibit the DN, thereby modulating these networks (Figure 2) (Zhou et al., 2022). Active tDCS parameters included a peak current of 1999 μA per electrode, culminating in a total current of 3,999 μA across the seven electrodes, with a 20-min stimulation duration that included 30-s ramp-up and ramp-down periods. Sham stimulation used the same electrode positions and current intensity as the DAN+/DN-tDCS, with current applied only during the first and last 30 s to simulate the sensation of real stimulation. The tDCS device was operated by personnel proficient in its use but not involved in the study, ensuring that neither the participants nor researchers knew the type of stimulation. After the intervention, participants completed the adverse event questionnaire to assess potential adverse effects (Woods et al., 2016), and they were asked to predict whether they received real or sham stimulation, to assess the blinding efficacy.

### 2.4 ANT

The ANT (Fan et al., 2002)^1^ was obtained from the Sackler Institute for Developmental Psychobiology and created using Java software. The test typically consists of a 24-trial practice with feedback and three 96-trial experimental blocks with no feedback. To enhance experiment efficiency and prevent mental fatigue, participants completed a practice and two experimental blocks, each lasting 6 min, with a 1-min rest between the two blocks. The test required participants to sit in front of a computer monitor and focus on a fixation cross (indicated by a cue and a target). When a target arrow appeared above or below the center fixation cross, they were required to provide a response as quickly as possible by pressing buttons on the keyboard corresponding to the direction of the target arrow (left or right) (Pauletti et al., 2017; De Souza Almeida et al., 2021). The ANT program recorded participants’ cognitive metrics automatically. Reaction times were measured to calculate the efficiency of the alerting, orienting, and executive networks using different conditions in each trial. The recorded values of two experimental blocks were averaged to determine the influence of the three attention network effects. The alerting effect refers to the ability to maintain vigilance, calculated as the difference in reaction time between no-cue and double-cue conditions. The orienting effect reflects the ability to select spatial information, calculated as the difference in reaction time between central-cue and spatial-cue conditions. The executive effect refers to the ability to deal with conflicting situations, calculated as the difference in reaction time between incongruent and congruent conditions (Posner and Petersen, 1990; Fan et al., 2002). Larger values of the alerting or orienting effect indicate stronger effects, demonstrating better performance in response to relevant cues. A lower value of the executive effect indicates better performance, reflecting quicker responses to conflict situations (Roy et al., 2015; Pauletti et al., 2017).

The primary outcome indicators of the study included the alerting, orienting, and executive effects. Additionally, reaction times for the six conditions and average accuracy for the entire ANT task were recorded. These conditions included no-cue, center-cue, double-cue, spatial-cue, incongruent, and congruent conditions.

### 2.5 Statistical analyses

To evaluate normal distribution, the data underwent the Shapiro–Wilk test. Non-normally distributed data were presented as the median (quartiles) using the non-parametric test, while normally distributed data were presented as the mean ± standard error using the *t*-test. Independent-sample t-tests were employed to evaluate differences in basic information between the two groups. To examine the effects of DAN+/DN-tDCS on ANT, independent samples t-tests were utilized to compare the absolute changes before and after stimulation to quantify between-group differences. Paired samples t-tests were used to compare within-group differences of the metrics. Furthermore, Fisher’s exact test and the Kruskal-Wallis test were employed to assess differences in blinding efficacy and adverse effect between the DAN+/DN-tDCS and sham groups. The significance threshold α was established at 0.05, with effect magnitudes quantified in terms of Cohen’s d. Statistical analyses were performed using SPSS version 27.0 (IBM Corp., Armonk, NY, United States).

## 3 Results

The study enrolled 40 participants, with 20 individuals in each group. All participants completed the experimental intervention and questionnaire (Table 1). Before the intervention, independent-sample *t*-tests revealed no significant differences in the baseline characteristics between the two groups (*p* > 0.05).

**Table 1:** Basic participant information.

Variables	DAN+/DN-tDCS (*n* = 20)	Sham (*n* = 20)	*t*	*p*
Age (years)	22.83 ± 2.26	22.13 ± 2.29	1.079	0.286
Females (%)	50.00	50.00		1.000^**X**^
Education (years)	16.83 ± 2.26	16.13 ± 2.29	1.737	0.087
Height (cm)	170.50 ± 8.88	171.92 ± 10.73	−0.498	0.621
Weight (kg)	63.42 ± 13.48	64.50 ± 12.65	−0.287	0.775

DAN+/DN-tDCS, excitatory frontoparietal network + inhibitory default network; DAN, dorsal attention network; DN, default network; Sham, sham stimulation; tDCS, transcranial direct current stimulation; ^**x**^ indicates categorical data compared using the chi-squared test.

### 3.1 Effects of DAN+/DN-tDCS on ANT performance

**Figure 3:**
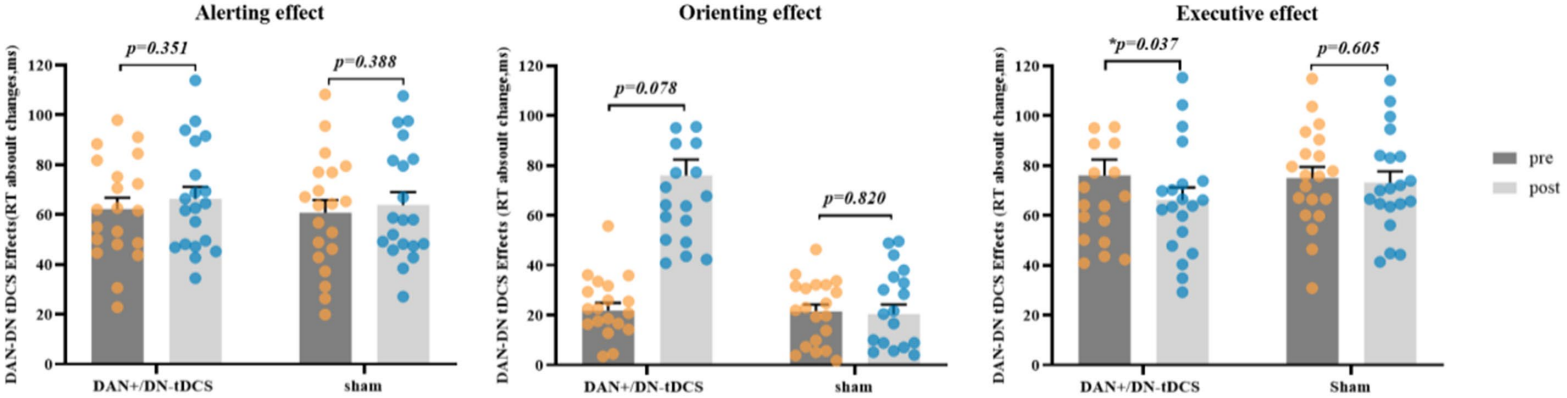
DAN+/DN-tDCS on alerting, orientastion, and executive effects. DAN+/DN-tDCS: excitatory frontoparietal network + inhibitory default network; sham: sham stimulus; pre: pre-test; post: post-test; *indicates a significant difference within the group. DAN, dorsal attention network; DN, default network; tDCS, transcranial direct current stimulation.

Paired-sample t-tests indicated that DAN+/DN-tDCS significantly enhanced executive effect performance (*t* = 2.245, *p* = 0.037, Cohen’s *d* = 0.502) and yielded marginally significant changes in orienting effect (*t* = −0.861, *p* = 0.078, Cohen’s *d* = −0.416). However, the three attention network effects showed no significant defferences in the sham group (*p >* 0.388) (Figure 3). Additionally, compared to that before stimulation, the DAN+/DN-tDCS group demonstrated a significant reduction in reaction times for the double cue, center cue, spatial cue, congruent cue, and incongruent cue conditions after stimulation (*p* < 0.05). The sham group showed significantly lower reaction times only for the spatial cue condition (*p* = 0.010) (Table 2). Furthermore, the average accuracy for judging target arrow direction in the ANT test significantly improved in the DAN+/DN-tDCS group after stimulation (*t* = −2.965; *p* = 0.008), while no significant changes were observed in the sham group.

**Table 2:** Reaction times for each stimulation group.

Variables	DAN+/DN-tDCS			Sham		
	Pre	Post	*p*	Pre	Post	*p*
No cue	468.77 ± 9.90	456.44 ± 7.20	0.058	488.36 ± 14.49	477.62 ± 10.24	0.317
Double cue	406.55 ± 11.23	390.10 ± 8.09	0.016^*^	424.00 ± 10.56	413.74 ± 8.19	0.129
Alerting effect	62.22 ± 4.55	66.33 ± 4.80	0.351	64.36 ± 6.44	63.89 ± 5.09	0.388
Central cue	411.21 ± 10.90	396.79 ± 8.01	0.016^*^	433.66 ± 11.00	421.17 ± 7.97	0.067
Spatial cue	396.30 ± 9.25	376.02 ± 8.21	0.002^*^	414.25 ± 11.07	401.31 ± 9.41	0.010^*^
Orienting effect	14.91 ± 3.12	21.80 ± 3.09	0.078	19.41 ± 3.96	17.71 ± 5.15	0.820
Congruent cue	400.67 ± 9.67	386.24 ± 7.36	0.027^*^	418.75 ± 11.66	407.39 ± 9.54	0.066
Incongruent cue	476.67 ± 13.10	452.44 ± 9.57	0.001^*^	493.76 ± 12.37	480.57 ± 8.55	0.067
Executive effect	76.00 ± 6.39	66.20 ± 4.99	0.037^*^	75.01 ± 4.45	73.18 ± 4.45	0.605

Reaction times (ms) are presented as the mean ± standard error categorized according to six cue types and three attentional effects. DAN+/DN-tDCS, excitatory frontoparietal network + inhibitory default network; DAN, dorsal attention network; DN, default network; Sham, sham stimulation; tDCS, transcranial direct current stimulation; *indicates a significant difference (*p* < 0.05).

Independent samples t-tests indicated no significant differences between the DAN+/DN-tDCS group and the sham group for the absolute changes in alerting, orienting, and executive effects (0.162 < *p <* 0.872). However, compared to the sham group, the DAN+/DN-tDCS group showed greater improvements in all three effects, with change values in orienting being 6.89 (Cohen’s d = −0.450) and executive effect being 9.81 (Cohen’s d = 0.451) (Table 3).

**Table 3:** The change values of network-based tDCS effects on alerting, orienting, and executive effect.

Variables	DAN+/DN-tDCS (Δ)	Sham (Δ)	Mean difference (95% confidence interval)	*p*	*Cohen’s d*
Alerting effect	4.11 ± 4.30	3.20 ± 3.62	0.91 (−10.46,12.29)	0.872	−0.051
Orienting effect	6.89 ± 3.70	−0.94 ± 4.07	7.83 (−3.31, 18.97)	0.163	−0.450
Executive effect	−9.81 ± 4.37	−1.84 ± 3.49	−7.97 (−19.29, 3.37)	0.162	0.451

Data is presented as the mean ± standard error. DAN+/DN-tDCS, excitatory frontoparietal network + inhibitory default network; DAN, dorsal attention network; DN, default network; Sham, sham stimulation; tDCS, transcranial direct current stimulation; Δ indicates the change values of network-based tDCS effects.

### 3.2 Blinding efficacy and adverse effects

Participants correctly guessed the type of stimulation with an accuracy rate of 52.50%; Fisher’s test showed *p* = 0.241, indicating a good blinding efficacy. The adverse event questionnaire revealed that the vast majority of all participants did not report severe discomfort during the experiment. However, significant differences were observed between the two groups in the severity of sensations such as stinging, itching, and skin reddening (*p* < 0.05) (Table 4). One participant in the DAN+/DN-tDCS group reported more severe itching, skin reddening, and fatigue (Table 4).

**Table 4:** Adverse effects.

Adverse effect	Grade	DAN+/DN-tDCS	Sham	*p*
Stinging	None	15.00	50.00	0.001*
	Mild	35.00	45.00	
	Moderate	50.00	5.00	
	Severe	0.00	0.00	
Itching	None	30.00	60.00	0.029*
	Mild	35.00	30.00	
	Moderate	30.00	10.00	
	Severe	5.00	0.00	
Burning	None	55.00	75.00	0.189
	Mild	25.00	15.00	
	Moderate	20.00	10.00	
	Severe	0.00	0.00	
Pain	None	30.00	65.00	0.051
	Mild	45.00	20.00	
	Moderate	25.00	15.00	
	Severe	0.00	0.00	
Skin reddening	None	45.00	95.00	<0.001*
	Mild	40.00	5.00	
	Moderate	10.00	0.00	
	Severe	5.00	0.00	
Fatigue	None	80.00	80.00	0.938
	Mild	15.00	20.00	
	Moderate	0.00	0.00	
	Severe	5.00	0.00	
Other	None	95.00	100.00	0.317
	Other discomfort	5.00	0.00	

DAN+/DN-tDCS, excitation of the dorsal attention network + inhibition of the default network; DAN, dorsal attention network; DN, default network; Sham, sham stimulation; tDCS, transcranial direct current stimulation. Data in the tables are percentages (%); * indicates a significant difference.

## 4 Discussion

This study aimed to explore the effects of network-based tDCS on attention function in healthy young adults. The results showed a significant reduction in reaction time on the ANT test and a significant enhancement in the executive network effect after DAN+/DN-tDCS. These findings suggested that the executive effect of attention function may be related to the coordinated activity between the DAN and the DN.

In this study, the DAN+/DN-tDCS protocol significantly enhanced executive effect in healthy adults. The ANT combines the cued reaction time test (Posner and Petersen, 1990) and the flanker task (Eriksen and Eriksen, 1974), measuring the efficiency of three subsystems of attention: alerting, orienting, and executive effect. The improvement in executive effect aligns with previous research findings, where the executive effect monitors and resolves task conflicts, such as error detection, decision-making, and planning (Fan et al., 2002; Callejas et al., 2005). The frontoparietal control network (FPCN) plays a crucial role in executive control, particularly in conflict resolution and task-switching processes (Zhao et al., 2019). Within the FPCN, there are two distinct subsystems with different functional connectivity patterns. FPCNA exhibits stronger connectivity with the DN than the DAN, while FPCNB shows the opposite pattern (Dixon, 2015; Spreng et al., 2016; Dixon et al., 2017). The DAN+/DN-tDCS protocol likely regulated the dynamic balance between the DN and DAN by flexibly coupling the FPCN with the connection patterns of the DN and DAN, thereby driving internal or external cognitive orientation. This flexible coupling may have enhanced the executive effect by optimizing the network’s ability to manage task conflict and improve performance on the ANT in healthy adults (Dixon et al., 2018).

Although the DAN+/DN-tDCS intervention did not significantly enhance the orienting effect, there was a trend of improvement (*t* = −0.861; *p* = 0.078), suggesting some modulatory efficiency on orienting effect. However, no significant difference was observed in the alerting effect after DAN+/DN-tDCS. Previous studies have reported that traditional tDCS targeting the right PPC significantly enhanced the orienting effect (Yin et al., 2012), indicating the positive role of the right PPC in orienting attention function. Yin et al. (2012) also found that the alerting effect was primarily linked to the right thalamus and supplementary motor regions, while the orienting effect was significantly correlated with the right parietal cortex, particularly the inferior parietal areas (Lo et al., 2019). Studies implicated that the alerting network may be associated with subcortical structures (the brainstem locus ceruleus) and circuits involving the right parietal and prefrontal cortex (Posner, 2008, 2012; Petersen and Posner, 2012). The DAN includes bilateral inferior parietal sulci, superior parietal lobules, and frontal eye fields, while the DN comprises the medial prefrontal cortex, precuneus, posterior cingulate cortex, and lateral parietal regions (Eryurek et al., 2022). Consequently, the activity of the DAN and the DN may not be the key neural mechanisms underlying the control of the alerting and orienting networks.

We observed a significant enhancement in average accuracy on the ANT task after DAN+/DN-tDCS. This finding may be associated with a learning effect induced by the repetitive nature of the ANT test the relative simplicity of the test tasks. Therefore, the significant improvement in average accuracy should be considered cautiously. Additionally, the timing of stimulation intervention and ANT test was self-determined by participants, which may have influenced their task performance.

This study has several limitations. First, the subjective judgment of electrical stimulation may significantly influence the results. During stimulation, participants may have subjectively assessed their received stimulation type, and their perceptions of skin sensations might enhance the placebo effect, improving performance in subsequent trials. Future studies should aim to optimize the stimulation protocol for the sham group, ensuring that both DAN+/DN-tDCS and sham stimulations elicit equivalent or similar somatosensory perceptions, thereby minimizing subjective biases among participants. Furthermore, this study employed a novel DAN+/DN-tDCS protocol targeting brain networks for modulation as our intervention approach. However, we did not incorporate a traditional tDCS protocol as a control to assess the disparities between the two methods. Consequently, we were unable to evaluate the superiority of the innovative network tDCS utilized in this investigation for modulating attention function. Lastly, the study only explored the immediate post-intervention effects of DAN+/DN-tDCS on attention function in healthy adults; further research is required to explore the long-term intervention or cumulative effects of this novel stimulation protocol and its potential for application in clinical populations.

## 5 Conclusion

The DAN+/DN-tDCS protocol may enhance attention function in healthy young adults, particularly showing improvement in executive effect. This study offers a novel intervention strategy for improving attention function based on brain network modulation. Attention function may rely on the functional activities of disparate brain networks. Therefore, establishing the relationships between different attention network effects and their corresponding brain networks in a rational and targeted manner is crucial for the effectiveness of stimulation protocols. Future studies should further explore the intervention effects of network-based tDCS on attention function in different populations and elucidate the potential underlying mechanisms to facilitate its widespread application in clinical rehabilitation.

## Data availability statement

The original contributions presented in the study are included in the article/supplementary material, further inquiries can be directed to the corresponding author.

## Ethics statement

The studies involving humans were approved by Institutional Review Board of the Shanghai University of Sports (102772020RT109). The studies were conducted in accordance with the local legislation and institutional requirements. The participants provided their written informed consent to participate in this study. Written informed consent was obtained from the individual(s) for the publication of any potentially identifiable images or data included in this article.

## Author contributions

XW: Writing – original draft, Writing – review & editing, Formal analysis, Validation. RZ: Data curation, Investigation, Writing – original draft. SZ: Data curation, Methodology, Software, Writing – original draft. YZ: Data curation, Investigation, Software, Supervision, Writing – original draft. XF: Data curation, Methodology, Writing – original draft. JL: Funding acquisition, Project administration, Resources, Supervision, Writing – original draft, Writing – review & editing.

## Footnotes

^1^ https://www.millisecond.com/download/library/ant/
